# Incremental Coding for Real-Time Remote Control over Bandwidth-Limited Channels and Its Applications in Smart Grids †

**DOI:** 10.3390/e26020122

**Published:** 2024-01-30

**Authors:** Yiyu Qiu, Junjie Wu, Wei Chen

**Affiliations:** 1 Department of Electronic Engineering, Tsinghua University, Beijing 100084, China; qiuyy22@mails.tsinghua.edu.cn (Y.Q.); wujj18@mails.tsinghua.edu.cn (J.W.); 2 Beijing National Research Center for Information Science and Technology (BNRist), Beijing 100084, China

**Keywords:** remote control over communication networks, industrial internet of things (IIoT), ultra-reliable and low-latency communications (URLLC), task-oriented communications

## Abstract

Remote control over communication networks with bandwidth-constrained channels has attracted considerable recent attention because it holds the promise of enabling a large number of real-time applications, such as autonomous driving, smart grids, and the industrial internet of things (IIoT). However, due to the limited bandwidth, the sub-packets or even bits have to be transmitted successively, thereby experiencing non-negligible latency and inducing serious performance loss in remote control. To overcome this, we introduce an incremental coding method, in which the actuator acts in real time based on a partially received packet instead of waiting until the entire packet is decoded. On this basis, we applied incremental coding to a linear control system to obtain a remote-control scheme. Both its stability conditions and average linear-quadratic-Gaussian-(LQG) cost are presented. Then, we further investigated a multi-user remote-control method, with a particular focus on its applications in the demand response of smart grids over bandwidth-constrained communication networks. The utility loss due to the bandwidth constraint and communication latency are minimized by jointly optimizing the source coding and real-time demand response. The numerical results show that the incremental-coding-aided remote control performed well in both single-user and multi-user scenarios and outperformed the conventional zero-hold control scheme significantly under the LQG metric.

## 1. Introduction

Remote control over communication networks is expected to play a key role in the future real-time applications of autonomous driving, the industrial internet of things (IIoT), and, especially, smart grids [1]. However, compared to remote-control systems [2] that use wired communications to provide real-time and reliable data transmission, remote control over wireless communication networks suffers serious communication limits [3], in terms of bandwidth, latency, and so on. Therefore, it is essential to study suitable technologies that effectively reduce the performance loss of remote control under these communication limits [4,5].

In particular, to satisfy the extremely strict low-latency communication requirement in the scenario of remote control over communication networks, the deep integration of communication and real-time control has been further considered. More specifically, the joint optimization of communication and some specific control processes were studied in [6,7,8,9,10,11,12,13,14,15]. Firstly, Tatikonda, Sahai, and Mitter studied the design of channel coding in [6,7,8] for a general distributed linear control system. They proposed a concept of “information complexity” to characterize the channel rate required for the stabilization of different control objectives. The trade-off between the communication rate and the linear-quadratic-regulator-(LQR) cost was studied in [9] for the linear stochastic system. In [10], the communication delay in a networked Markov decision process was studied. The performance of such networked Markov decision processes was also shown to be extremely sensitive to communication latency. In the quantized linear-quadratic-Gaussian-(LQG) control problem, a lack of separation principle was found in [11]. It was shown that feedback control, estimation, and quantization cannot be separated. In [12], goal-oriented quantization was considered for practical control tasks, in which quantization policy and decision making were jointly optimized. In [13], finite-blocklength coding was jointly considered in a wireless networked control system (WNCS). The counterintuitive result found was that a scalar linear control system is stabilized with an arbitrarily large latency when the SNR of the wireless channel is high enough. In [14], a coding-free control method was proposed in WNCS, which aimed to minimize the sum control cost of multiple plants by allocation of data-transmission power. A differential coding scheme was proposed in [15], based on which the stability properties were analyzed for networked controlled linear systems. Furthermore, the effect of quantization and estimation on the stability of linear control systems has also been widely addressed, such as in quantization accuracy [16], the quantization scheme [17,18], and state estimation [19,20].

Communication-and-control co-design is particularly essential to the application of demand response in smart grids. More specifically, demand response alters the user’s demand profile effectively to make it match the electricity supply, which is important to improve the reliability of smart grid operation [21]. However, the smooth implementation of demand response requires the guarantee of extremely low communication latency [22]. The millisecond-level delay of communication may induce a certain degree of performance loss which cannot be ignored in demand response. To fulfill the extremely low latency requirement [23], the deep integration of communication and smart grids needed to be further considered. In the demand-response scenario of smart grids, the communication requirements were thoroughly studied in [24,25,26]. The wireless-communication-bandwidth requirement for demand response was studied in [24]. It was shown that the wireless bandwidth for demand response can be extremely limited as the number of users in the smart grid increases. On the other hand, with a larger number of users, demand response will become more effective [25]. Therefore, a highly scalable communication system for demand response is particularly important. Furthermore, the quality-of-service-(QoS) requirements on data transmission for real-time sensing and metering were also studied in [26]. It was shown that for the smooth implementation of various pricing schemes in demand response, the communication latency of real-time metering is required to be a few milliseconds, which is extremely strict.

The motivation for incremental coding is to achieve optimal control based on partially received packets. Similar ideas can also be found in progressive coding in image- and video-transmission problems. For example, the wavelet-transform-based method [27] is widely used in the JPEG2000. Furthermore, multiple-description coding [28] can optimize the decoding results based on each individually received data packet under unreliable channel conditions. Layered coding, on the other hand, can achieve rate scalability by providing layered embedded bit streams that can be decoded at different bit rates [29]. A closer example of incremental decoding appeared in [30], where an iterative-quantization algorithm was proposed for quantized Kalman filtering under communication-limited conditions. However, partial decoding of data packets was not considered.

In our previous work [31,32,33,34], we proposed an incremental-coding-based communication mechanism for real-time control tasks to satisfy the extremely strict requirement of communication latency, in which the source-codebook design and control process were jointly optimized. More specifically, to minimize the performance loss induced by information latency in real-time control or decision making, the incremental-coding mechanism allows the controller to take action in real time based on the currently received partial codeword instead of waiting for the entire codeword. On this basis, joint optimization of source coding and decision making was finally formulated as a general problem and solved by a dynamic-programming algorithm. However, to broaden the application scope of these works, we also needed to extend our control model to linear control systems, which are widely implemented in industrial applications, such as the demand response of smart grids.

For this paper, we focused on introducing an incremental-coding-based communication mechanism into linear control systems. More specifically, we first studied the scenario of a linear control system with a single controller and a single plant. In this scenario, an incremental-coding-based control scheme was proposed, based on which we compared LQG control performance to that of a traditional zero-hold control scheme. Then, the necessary and sufficient conditions for the plant to be stable under these two control schemes were derived, in terms of wireless bandwidth and source-code length, respectively. The analytical results showed that the stabilization conditions under these two control schemes were the same. In other words, the incremental-coding-based control scheme showed no performance gain on the stabilization of the plant, compared to the traditional zero-hold control scheme. On the other hand, the LQG performance measures of these two control schemes were further compared. In particular, the respective achievable upper bounds of the average LQG control cost under each of these two control schemes were derived. The analytical results show that the incremental-coding-based scheme significantly outperformed the zero-hold scheme under the LQG performance measure. On this basis, we extended the linear control system to the case of a multi-user control scenario. A specific multi-user control scenario for demand-response management (DRM) of smart grids was studied. In this scenario, the cumulative mean square error (MSE) between the electricity load and the supply was formulated as the LQG control cost. Then, both the centralized direct control and the distributed control through electricity price were considered in terms of DRM. The minimization of performance loss induced by the latency of the demand-scheduling signaling was finally achieved by introducing the incremental-coding-based communication mechanism with joint optimization of source coding and real-time decision making. Finally, the numerical results also showed the potential of introducing the incremental-coding mechanism in DRM.

The remainder of this paper is organized as follows. In Section 2, we introduce the model of the discrete-time scalar linear control system, along with the corresponding quantization and source-coding schemes. In Section 3, we propose the incremental-coding-based control scheme and compare its LQG control performance to that of the traditional zero-hold control scheme. In Section 4, we extend the linear control system to a specific multi-user control scenario in the DRM of the smart grid. Section 5 demonstrates our conclusions with numerical results. Finally, Section 6 concludes the paper.

## 2. System Model

In this section, we first introduce the composition and time discretization of a linear-time-invariant-(LTI) discrete-time control system. To satisfy the growing demand for the application of wireless networked control, the actuator and sensor are assumed to be separated and connected over a wireless channel with limited bandwidth. We then introduce the quantization and coding scheme of such a communication system with a limited bandwidth.

### 2.1. Discrete-Time Linear Control System

**Figure 1:**
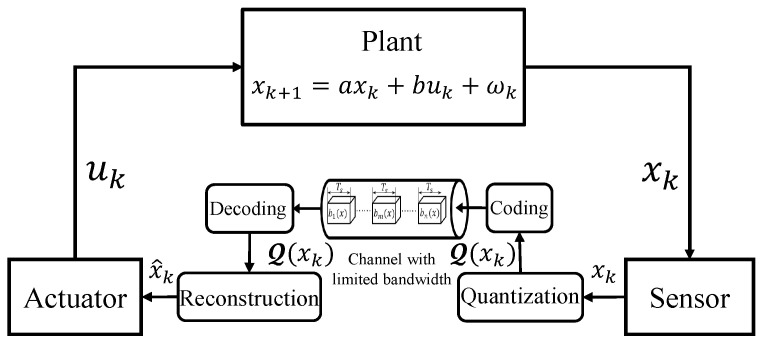
System Model.

As shown in Figure 1, we consider a scalar linear control system consisting of a plant, an actuator, and a sensor. More specifically, the plant denotes the practical dynamical physical environment. The evolution of state x(t) in the plant can be described by a linear differential equation as follows:

(1) $$\dot{x}\left(t\right)=a_{c}x\left(t\right)+b_{c}u\left(t\right)+w\left(t\right),\;\;\forall \;t\in \left[0,+\infty \right),$$

where x(t) is the state of the plant and variable $\dot{x}\left(t\right)$ is differentiation of x(t) in the time domain. Variable u(t) is the control action performed by the actuator, and variable $w\left(t\right)\in \left[-w_{0},w_{0}\right]$ is a bounded stochastic disturbance with zero mean in the state-evolution process. Parameters $a_{c}$ and $b_{c}$ are positive constants that depend on the specific dynamical physical environment. The initial state x(0) belongs to a bounded set $\left[-x_{0,max},x_{0,max}\right]$. With the information of state x(t) the actuator can perform appropriate u(t) to make state x(t) stable (approach zero).

To facilitate the design of the actuator, time is discretized into periods with duration $T_{s}$. In each period, the actuator performs the constant control action, i.e.,

(2) $$u\left(t\right)=u_{k},\;\forall \;kT_{s}\leq t<\left(k+1\right)T_{s}.$$

On this basis, let $x_{k}$ denote the state of the plant at the beginning of *k*-th period, i.e., $x_{k}=x\left(kT_{s}\right)$. Then, the state evolution of the plant can be expressed in the following discrete-time form:

(3) $$x_{k+1}=ax_{k}+bu_{k}+w_{k},\;\;\forall \;k\in N,$$

where $x_{0}=x\left(0\right)\in \left[-x_{0,max},x_{0,max}\right]$. Furthermore, the parameters *a*, *b*, and the stochastic disturbance $w_{k}$ in Equation (3) depend on the parameters $a_{c}$, $b_{c}$ in Equation (1) and the duration $T_{s}$ of the control periods. By analyzing the linear differential equation [35], we can establish that

(4) $$\begin{array}{cc} a & =e^{a_{c}T_{s}},\\ b & =\frac{b_{c}}{a_{c}}\left(e^{a_{c}T_{s}}-1\right),\\ w_{k} & =\int_{kT_{s}}^{\left(k+1\right)T_{s}}e^{a_{c}\left(\left(k+1\right)T_{s}-\tau \right)}w\left(\tau \right)d\tau . \end{array}$$

As w(τ) in Equation (4) is a bounded stochastic disturbance with zero mean, such that $w\left(\tau \right)\in \left[-w_{0},w_{0}\right]$, we can deduce that $w_{k}\in \left[-w_{max},w_{max}\right]$ is also a bounded stochastic disturbance with zero mean, where $w_{max}=a_{c}^{-1}\left(e^{a_{c}T_{s}}-1\right)w_{0}$.

We assume that the actuator cannot obtain the state of the plant by itself. To overcome this, the sensor measures the state of the plant and transmits it to the actuator periodically through a wireless channel with limited bandwidth. The measurement process of the sensor is assumed to be perfect, in which the signal distortion and the time consumption caused by the measurement can be negligible. The time interval between two measurements of the sensor is $nT_{s}$. Therefore, the actuator can obtain the information of $x_{kn},\;\forall k\in N$ from the measurement of the sensor. Based on the information of $x_{kn}$, the actuator decides the control action $u_{i}$ for any i=kn,kn+1,⋯,(k+1)n−1. Based on the state evolution of the plant given in Equation (3), the state in the k+1-th measurement $x_{(k+1)n}$ has the following relationship with the state in the *k*-th measurement $x_{kn}$:

(5) $$x_{(k+1)n}=a^{n}x_{kn}+\sum_{i=0}^{n-1}a^{n-1-i}bu_{kn+i}+\sum_{i=0}^{n-1}a^{n-1-i}w_{kn+i}.$$

### 2.2. Quantization and Source-Coding Schemes

The sensor measures the state of the plant with period $nT_{s}$ and transmits it to the actuator through a wireless channel with bandwidth *W*. In particular, the data transmission of the sensor uses BPSK modulation with the symbol period 1/W, i.e., one bit is transmitted every 1/W seconds [36]. Furthermore, to align the temporal granularity of the control action and the information transmission, the duration of the control period is consistent with the symbol period, $T_{s}=1/W$. As a result, the sensor can transmit n−1 bits during the time interval between two measurements.

Before the *k*-th data transmission, the analog state $x_{kn}$ of the plant is quantified by a $2^{n-1}$-step uniform quantizer $Q_{H_{k}}$, which is given by

(6) $$Q_{H_{k}}\left(x\right)=\left\{\begin{array}{ccc} \left(\left\lceil \frac{x}{\Delta }\right\rceil -\frac{1}{2}\right)\Delta ,\; & \; & 0<x\leq H_{k},\\ \left(\left\lfloor \frac{x}{\Delta }\right\rfloor +\frac{1}{2}\right)\Delta , & \; & -H_{k}<x\leq 0,\\ 0, & \; & else, \end{array}\right.$$

where $H_{k}$ is the range of the analog state, i.e., $x_{kn}\in \left[-H_{k},H_{k}\right]$ and the variable $\Delta =\frac{H_{k}}{2^{n-2}}$ is the quantized interval. After quantizing the analog state into a discrete value, the sensor implements a source codebook to encode the discrete-state value $Q_{H_{k}}\left(x\right)$ into the codeword $c_{x}$ with n−1 bits. For such a uniform quantizer $Q_{H_{k}}$, the mapping between the analog state *x* and the codeword $c_{x}$ is given as follows:

(7) $$c_{x}\left(i\right)=\left\{\begin{array}{ccc} I\{x>0\}, & \; & i=1,\\ \left\lfloor \frac{|Q_{H_{k}}\left(x\right)|}{2^{n-1-i}\Delta }\right\rfloor \;\mathrm{mod}\;2, & \; & 2\leq i\leq n-1. \end{array}\right.$$

**Figure 2:**
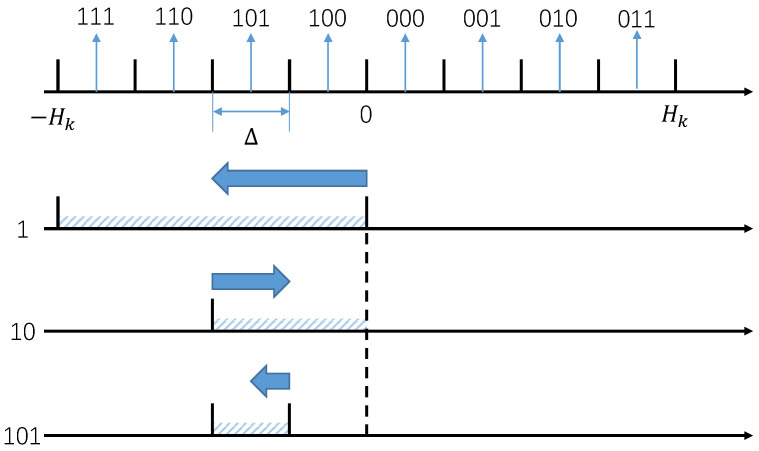
Example of quantization and coding when measurement period n=4. The black dashed line represents the position of the origin corresponding to x=0, the blue shaded areas represent the range of values *x* based on the current reception, and the blue arrows indicate the change in the estimate of *x* after each bit is received.

For ease of understanding, we present the example of quantization and source coding in the case of n=4, as shown in Figure 2. From this example, we can see that the range of the uncertainty region of the analog state *x* will be reduced to exactly half of the original uncertainty region when one new bit of codeword $c_{x}$ is received at the actuator. As a result, when part of codeword $c_{x}$ is received, the actuator can extract the information of analog state *x* immediately for real-time control instead of waiting until the complete codeword is received. This new way of information utilization, named “incremental coding” [32], may improve the performance of delay-critical linear control systems substantially.

In the following, we shall compare the incremental-coding-based control scheme and the traditional zero-hold control scheme under the LQG performance measure based on the above wireless networked linear control system. In particular, such a comparison of the LQG performance measure is also extended to multi-user control scenarios.

## 3. Incremental-Coding-Empowered Linear Feedback Systems

In this section, we propose the incremental-coding-based control scheme and compare it to the traditional zero-hold control scheme. More specifically, under these control schemes, the stability domain of the plant is obtained in the two-dimensional space of wireless bandwidth and source-code length. On this basis, we further compare the LQG performance measure of these control schemes. The analytical results show that the incremental-coding-based scheme outperforms the zero-hold scheme significantly under the LQG performance measure.

### 3.1. Zero-Hold Control Scheme

In this subsection, we introduce the traditional zero-hold control scheme, in which the actuator controls the plant only when the codeword $c_{x}$ is fully decoded. In other words, the actuator keeps waiting until the complete information of $Q_{H_{k}}\left(x\right)$ is received. Under the LQG manner, the control input $u_{kn+i},\;\;i=1,2,\cdots ,n-1$ of the zero-hold scheme is given as follows:

(8) $$u_{kn+i}=\left\{\begin{array}{ccc} 0, & \; & 0\leq i\leq n-2,\\ -\frac{a^{n}}{b}Q_{H_{k}}\left(x_{kn}\right), & \; & i=n-1. \end{array}\right.$$

The result of control action $u_{(k+1)n-1}$ in the last control period can be derived, based on Equation (5). More specifically, to make $x_{(k+1)n}$ approach zero as much as possible, we should maintain that

(9) $$\sum_{i=0}^{n-1}a^{n-1-i}bu_{kn+i}=-a^{n}x_{kn}.$$

As $u_{kn+i}=0,\forall \;0\leq i\leq n-2$, when the actuator obtains the complete information of $Q_{H_{k}}\left(x_{kn}\right)$ in the last control period, the control action should be $u_{(k+1)n-1}=-\frac{a^{n}}{b}Q_{H_{k}}\left(x_{kn}\right)$.

Under the control action $u_{kn+i}$ given in Equation (8), the analytical results of state $x_{kn+i}$ between the *k*-th measurement and the k+1-th measurement are obtained as follows:

(10) $$x_{kn+i}=\left\{\begin{array}{ccc} a^{i}x_{kn}+\sum_{l=0}^{i-1}a^{i-1-l}w_{kn+l}, & \; & 1\leq i\leq n-1,\\ a^{n}\left(x_{kn}-Q_{H_{k}}\left(x_{kn}\right)\right)+\sum_{l=0}^{n-1}a^{n-1-l}w_{kn+l}, & \; & i=n. \end{array}\right.$$

The result of Equation (10) shows that the value of state $x_{kn+i}$ is divided into two parts. The first part is induced by the state of the *k*-th measurement $x_{kn}$. The second part is induced by the stochastic disturbance in the state-evolution process, which is inevitable. Fortunately, the value of the first part can be decreased significantly once the complete information of $Q_{H_{k}}\left(x_{kn}\right)$ is obtained in the last control period. This is because based on Equation (6) we obtain

(11) $$-\frac{H_{k}}{2^{n-1}}\leq x_{kn}-Q_{H_{k}}\left(x_{kn}\right)\leq \frac{H_{k}}{2^{n-1}},$$

so that the bound is much smaller than that of original bound on $x_{kn}$, i.e., $-H_{k}\leq x_{kn}\leq H_{k}$.

**Lemma 1.**  *Under the zero-hold control scheme, the quantization-interval size $H_{k+1}$ in the k+1-th measurement has the following recursive relationship with $H_{k}$ in the k-th measurement:*

(12) $$H_{k+1}=\frac{a^{n}}{2^{n-1}}H_{k}+\frac{a^{n}-1}{a-1}w_{max}.$$

**Proof.**  Based on the analytical results of state $x_{kn+i}$ in Equation (10), the state of the plant at the k+1-th measurement is given as follows:

(13) $$x_{(k+1)n}=a^{n}\left(x_{kn}-Q_{H_{k}}\left(x_{kn}\right)\right)+\sum_{l=0}^{n-1}a^{n-1-l}w_{kn+l}.$$

As $-w_{max}\leq w_{kn+l}\leq w_{max}$, then we have

(14) $$-\frac{a^{n}-1}{a-1}w_{max}\leq \sum_{l=0}^{n-1}a^{n-1-l}w_{kn+l}\leq \frac{a^{n}-1}{a-1}w_{max}.$$

Substituting inequality (11) and (14) into Equation (13), we can finally ascertain that

(15) $$\begin{array}{cc} x_{(k+1)n} & \geq -\left(\frac{a^{n}}{2^{n-1}}H_{k}+\frac{a^{n}-1}{a-1}w_{max}\right) \end{array}$$

(16) $$\begin{array}{cc} x_{(k+1)n} & \leq \frac{a^{n}}{2^{n-1}}H_{k}+\frac{a^{n}-1}{a-1}w_{max}. \end{array}$$

□

### 3.2. Incremental-Coding-Based Control Scheme

In this subsection, we shall introduce the incremental-coding-based control scheme. By contrast with the zero-hold control scheme, the incremental-coding based-control scheme extracts the information of the state in real time based on the currently received partial codeword instead of waiting for the complete codeword.

Let ${\hat{x}}_{kn}\left(kn+i\right)$ denote the estimation of state $x_{kn}$ in the kn+i-th control period based on the incremental decoding result under the given codebook in Equation (7). By taking the center point of the uncertainty region as the estimation value (this is reasonable when state $x_{kn}$ follows a uniform distribution in an uncertainty region), we have

(17) $$\begin{array}{c} {\hat{x}}_{kn}\left(kn+i\right)=\left\{\begin{array}{ccc} \left(\left\lceil \frac{x_{kn}2^{i-1}}{H_{k}}\right\rceil -\frac{1}{2}\right)\frac{H_{k}}{2^{i-1}}, & \; & 0\leq x_{kn}\leq H_{k},\\ \left(\left\lfloor \frac{x_{kn}2^{i-1}}{H_{k}}\right\rfloor +\frac{1}{2}\right)\frac{H_{k}}{2^{i-1}}, & \; & -H_{k}\leq x_{kn}<0, \end{array}\right. \end{array}$$

where i=1,2,⋯,n−1 and ${\hat{x}}_{kn}\left(kn\right)=0$ in the case of i=0.

Let V(kn+i) denote the estimation error of state $x_{kn}$ in the kn+i-th control period, i.e., $V\left(kn+i\right)=x_{kn}-{\hat{x}}_{kn}\left(kn+i\right)$. Then, based on ${\hat{x}}_{kn}\left(kn+i\right)$ given in Equation (17), the estimation error V(kn+i) is bounded by *i* as

(18) $$-\frac{H_{k}}{2^{i}}\leq V\left(kn+i\right)\leq \frac{H_{k}}{2^{i}}.$$

As a result, the estimation error decreases exponentially as *i* increases. Using the estimation of state $x_{kn}$ under the incremental-coding-based communication mechanism, the actuator adjusts control action $u_{kn+i}$ in real time to make the state approach zero as closely as possible. To this end, the control action under the incremental-coding-based scheme is given by

(19) $$\begin{array}{c} u_{kn+i}=\frac{a^{i+1}}{b}\left({\hat{x}}_{kn}\left(kn+i-1\right)-{\hat{x}}_{kn}\left(kn+i\right)\right),\;\forall \;i=1,2,\cdots ,n-1, \end{array}$$

where $u_{kn}=0$, because no information of $x_{kn}$ is received in the kn-th control period.

**Lemma 2.**  *Under the incremental-coding-based control scheme, the state of the plant can be presented as the following general-term formula:*

(20) $$x_{kn+t}=a^{t}V\left(kn+t-1\right)+\sum_{l=0}^{t-1}a^{t-1-l}w_{kn+l},\;\forall \;t=1,2,\cdots ,n.$$

**Proof.**  The proof of Lemma 2 is based on mathematical induction. We assume that state $x_{kn+t}$ obeys the form presented in Equation (20) when t=i. We then show that $x_{kn+t}$ still obeys the form presented in Equation (20) when t=i+1. More specifically, substituting control action $u_{kn+i}$ given by Equation (19) into the state evolution of the plant given by Equation (3), we can ascertain that

(21) $$\begin{array}{cc} x_{kn+i+1} & =ax_{kn+i}+bu_{kn+i}+w_{kn+i}\\ \; & =a^{i+1}\left(x_{kn}-{\hat{x}}_{kn}\left(kn+i-1\right)\right)+\sum_{l=0}^{i-1}a^{i-l}w_{kn+l}\\ \; & \;+a^{i+1}\left({\hat{x}}_{kn}\left(kn+i-1\right)-{\hat{x}}_{kn}\left(kn+i\right)\right)+w_{kn+i}\\ \; & =a^{i+1}V\left(kn+\left(i+1\right)-1\right)\\ \; & \;+\sum_{l=0}^{(i+1)-1}a^{(i+1)-1-l}w_{kn+l}. \end{array}$$

The result of Equation (21) shows that if state $x_{kn+t}$ obeys Equation (20) when t=i, then it still obeys Equation (20) when t=i+1. Fortunately, by substituting control action $u_{kn}=0$ into the state evolution of the plant, we have

(22) $$x_{kn+1}=aV\left(kn\right)+w_{kn}.$$

As a result, the form presented in Equation (20) is established when t=1. On this basis, the form presented in Equation (20) is also established when t=2,3,⋯,n. □

Comparing the results of Equations (10) and (20), we note that the state of the plant under the incremental-coding-based control scheme is smaller than that under the zero-hold control scheme. In particular, based on the analytical result in Equation (20), the value of the state at the k+1-th measurement is obtained as

(23) $$x_{(k+1)n}=a^{n}\left(x_{kn}-Q_{H_{k}}\left(x_{kn}\right)\right)+\sum_{l=0}^{n-1}a^{n-1-l}w_{kn+l},$$

which is exactly the same as $x_{(k+1)n}$ under the zero-hold control scheme shown in Equation (10). As a result, when we implement the incremental-coding-based control scheme, the quantization interval size $H_{k+1}$ and $H_{k}$ have the same recursive relationship as shown in Lemma 1, i.e.,

(24) $$H_{k+1}=\frac{a^{n}}{2^{n-1}}H_{k}+\frac{a^{n}-1}{a-1}w_{max}.$$

### 3.3. Stability-Analysis-and-LQG-Performance Comparison

Based on the analytical result of the state given in Equations (10) and (20), we can further study the necessary and sufficient conditions for the stabilization of the plant under the zero-hold control scheme and the incremental-coding-based control scheme, respectively. In particular, the stabilization condition of the plant in the mean-square sense can be defined as

(25) $$\underset{t\to \infty }{lim sup}E(|x_{t}{|}^{2})<\infty .$$

Under the LQG control manner, we can further define the square of the state as the LQG control cost. As a result, the average control cost $C_{ave}$ is given by

(26) $$\begin{array}{cc} C_{ave} & =\lim_{T\to \infty }\frac{1}{T}\sum_{t=0}^{T}E(|x_{t}{|}^{2}),\\ \; & =\lim_{L\to \infty }\frac{1}{L}\sum_{k=1}^{L}\frac{1}{n}\sum_{t=kn+1}^{(k+1)n}E(|x_{t}{|}^{2}),\\ \; & =\lim_{L\to \infty }\frac{1}{L}\sum_{k=1}^{L}C_{ave}^{\left(k\right)}, \end{array}$$

where $C_{ave}^{\left(k\right)}=\frac{1}{n}\sum_{t=kn}^{(k+1)n-1}E(|x_{t}{|}^{2})$ is the average cost between the *k*-th measurement and the k+1-th measurement. To ensure the stabilization condition listed in condition (25), we only need to ensure that the average LQG control cost $C_{ave}$ in Equation (26) is bounded.

**Lemma 3.**  *Under the zero-hold control scheme, the average cost between the k-th measurement and the k+1-th measurement, i.e., $C_{ave}^{\left(k\right)}$ is bounded by $H_{k}$ and $w_{max}$ as follows:*

(27) $$C_{ave}^{\left(k\right)}\leq \frac{a^{2n}-1}{n(a^{2}-1)}H_{k}^{2}+\left(\frac{a^{2n}-1}{n(a^{2}-1)}-\frac{1}{a^{2}-1}\right)\sigma_{w}^{2},$$

*where $\sigma_{w}^{2}$ is the variance of stochastic disturbance $w_{k}$. The same holds for (27) when $x_{kn}=\pm H_{k}$, and $w_{kn+i}=\pm w_{max},\;\forall \;i=0,1,\cdots ,n-1$.*

**Proof.**  Based on Equation (26) and the analytical results of state $x_{kn+i}$ in Equation (10), the average LQG control cost $C_{ave}^{\left(k\right)}$ under the zero-hold control scheme is given by Equation (28):

(28) $$\begin{array}{cc} \frac{1}{n}\sum_{t=0}^{n-1}E(|x_{kn+t}{|}^{2}) & =\frac{1}{n}\sum_{t=0}^{n-1}E\left(a^{2t}x_{kn}^{2}+{\left(\sum_{l=0}^{t-1}a^{t-1-l}w_{kn+l}\right)}^{2}+2a^{t}x_{kn}\left(\sum_{l=0}^{t-1}a^{t-1-l}w_{kn+l}\right)\right)\\ \; & \overset{\left(I\right)}{=}\frac{1}{n}\sum_{t=0}^{n-1}E\left(a^{2t}x_{kn}^{2}+\sum_{l=0}^{t-1}a^{2(t-1-l)}w_{kn+l}^{2}\right)\\ \; & \overset{\left(\mathrm{II}\right)}{\leq }\frac{1}{n}\sum_{t=0}^{n-1}\left(a^{2t}H_{k}^{2}+\sum_{l=0}^{t-1}a^{2(t-1-l)}\sigma_{w}^{2}\right)\\ \; & =\frac{a^{2n}-1}{n(a^{2}-1)}H_{k}^{2}+\left(\frac{a^{2n}-1}{n(a^{2}-1)}-\frac{1}{a^{2}-1}\right)\sigma_{w}^{2}. \end{array}$$

More specifically, equation (I) holds in Equation (28) because the stochastic disturbance $w_{kn+i}$ is zero mean and independent of the state at the *k*-th measurement $x_{kn}$. Inequality (II) holds in Equation (28) because $x_{kn}\in \left[-H_{k},H_{k}\right]$ and $w_{kn+l}\in \left[-w_{max},w_{max}\right]$. Therefore, the same holds in (27) when $x_{kn}=\pm H_{k}$ and $w_{kn+i}=\pm w_{max},\;\forall \;i=0,1,\cdots ,n-1$. □

**Lemma 4.**  *Under the incremental-coding-based control scheme, the expectation of average cost between the k-th measurement and the k+1-th measurement, i.e., $C_{ave}^{\left(k\right)}$ is bounded by $H_{k}$ and $w_{max}$ as follows:*

(29) $$C_{ave}^{\left(k\right)}\leq \frac{a^{2n}-4^{n}}{n4^{\left(n-1\right)}\left(a^{2}-4\right)}H_{k}^{2}+\left(\frac{a^{2n}-1}{n(a^{2}-1)}-\frac{1}{a^{2}-1}\right)\sigma_{w}^{2}.$$

*The same holds in (29) when $x_{kn}=\pm H_{k}$ and $w_{kn+i}=\pm w_{max},\;\forall \;i=0,1,\cdots ,n-1$.*

**Proof.**  According to Lemma 2 and Equation (26), the average LQG control cost $C_{ave}^{\left(k\right)}$ under the incremental-coding-based control scheme is given by Equation (30):

(30) $$\begin{array}{cc} \frac{1}{n}\sum_{t=0}^{n-1}E(|x_{kn+t}{|}^{2}) & =\frac{1}{n}E(\sum_{t=1}^{n-1}(a^{2t}V^{2}\left(kn+t-1\right)+{\left(\sum_{l=0}^{t-1}a^{\left(t-1-l\right)}w_{kn+l}\right)}^{2}\\ \; & \;+2a^{t}V\left(kn+t-1\right)\left(\sum_{l=0}^{t-1}a^{\left(t-1-l\right)}w_{kn+l}\right))+{\left|x_{kn}\right|}^{2})\\ \; & \overset{\left(I\right)}{=}\frac{1}{n}E\left(\sum_{t=1}^{n-1}\left(a^{2t}V^{2}\left(kn+t-1\right)+\sum_{l=0}^{t-1}a^{2(t-1-l)}w_{kn+l}^{2}\right)+{\left|x_{kn}\right|}^{2}\right)\\ \; & \overset{\left(\mathrm{II}\right)}{\leq }\frac{1}{n}\sum_{t=0}^{n-1}\left(a^{2t}\frac{H_{k}^{2}}{2^{2t}}+\sum_{l=0}^{t-1}a^{2(t-1-l)}\sigma_{w}^{2}\right)\\ \; & =\frac{a^{2n}-4^{n}}{n4^{\left(n-1\right)}\left(a^{2}-4\right)}H_{k}^{2}+\left(\frac{a^{2n}-1}{n(a^{2}-1)}-\frac{1}{a^{2}-1}\right)\sigma_{w}^{2}. \end{array}$$

Similar to the proof of Lemma 3, equation (I) holds in Equation (30) because the stochastic disturbance $w_{kn+i}$ is zero mean and independent of the estimation error V(kn+t−1). Inequality (II) holds in Equation (30) because $x_{kn}\in \left[-H_{k},H_{k}\right]$ and $w_{kn+l}\in \left[-w_{max},w_{max}\right]$. Therefore, the same holds in (29) when $x_{kn}=\pm H_{k}$ and $w_{kn+i}=\pm w_{max},\;\forall \;i=0,1,\cdots ,n-1$. □

**Corollary 1.**  *The achievable upper bound of the average LQG control cost $C_{ave}^{\left(k\right)}$ between the k-th measurement and the k+1-th measurement under the incremental-coding-based control scheme outperforms that under the zero-hold control policy.*

**Proof.**  Based on Equations (28) and (30), we have

$$\begin{array}{cc} \; & \;\left(\frac{a^{2n}-4^{n}}{n4^{\left(n-1\right)}\left(a^{2}-4\right)}H_{k}^{2}+\left(\frac{a^{2n}-1}{n(a^{2}-1)}-\frac{1}{a^{2}-1}\right)\sigma_{w}^{2}\right)\\ \; & \;-\left(\frac{a^{2n}-1}{n(a^{2}-1)}H_{k}^{2}+\left(\frac{a^{2n}-1}{n(a^{2}-1)}-\frac{1}{a^{2}-1}\right)\sigma_{w}^{2}\right)\\ \; & =\frac{1}{n}\sum_{t=0}^{n-1}\left(a^{2t}\frac{H_{k}^{2}}{2^{2t}}+\sum_{l=0}^{t-1}a^{2(t-1-l)}\sigma_{w}^{2}\right)-\frac{1}{n}\sum_{t=0}^{n-1}\left(a^{2t}H_{k}^{2}+\sum_{l=0}^{t-1}a^{2(t-1-l)}\sigma_{w}^{2}\right)\\ \; & =\frac{H_{k}^{2}}{n}\sum_{t=0}^{n-1}\left({\left(\frac{a^{2}}{4}\right)}^{t}-{\left(a^{2}\right)}^{t}\right)\leq 0. \end{array}$$

As $\left({\left(\frac{a^{2}}{4}\right)}^{t}-{\left(a^{2}\right)}^{t}\right)\leq 0$ for any t=0,1,⋯,n−1, we have $\sum_{t=0}^{n-1}\left({\left(\frac{a^{2}}{4}\right)}^{t}-{\left(a^{2}\right)}^{t}\right)\leq 0$. □

**Theorem 1.**  *The necessary and sufficient condition for the stability of the plant in relation to both the wireless bandwidth W and the source-code length n are as follows:*

(31) $$e^{\frac{na_{c}}{W}}-2^{n-1}<0,$$

*regardless of whether the zero-hold control scheme or the incremental-coding-based control scheme is implemented.*

**Proof.**  According to Equation (26), the necessary and sufficient condition in Equation (25) for the stability of the plant is equivalent to ensure that the average LQG control cost $C_{ave}^{\left(k\right)}$ is bounded for any *k*. Based on Lemmas 3 and 4, we note that the achievable upper bound of $C_{ave}^{\left(k\right)}$ increases linearly with $H_{k}^{2}$ and $w_{max}^{2}$. As $w_{max}=a_{c}^{-1}\left(e^{a_{c}T_{s}}-1\right)w_{0}$ is bounded, the necessary and sufficient condition in Equation (25) is thereby equivalent to ensure that $H_{k}$ is bounded for any positive integer *k*. On the other hand, the update of $H_{k}$ is given by

(32) $$H_{k+1}=\frac{a^{n}}{2^{n-1}}H_{k}+\frac{a^{n}-1}{a-1}w_{max},$$

regardless of whether the zero-hold control scheme or the incremental-coding-based control scheme is implemented. Therefore, the condition of bounded $H_{k}$ for any positive integer *k* is equivalent to the condition $\frac{a^{n}}{2^{n-1}}<1$. By substituting $a=e^{a_{c}T_{s}}$ and $T_{s}=\frac{1}{W}$ into $\frac{a^{n}}{2^{n-1}}<1$, we obtain $e^{\frac{na_{c}}{W}}-2^{n-1}<0$ as the necessary and sufficient condition for the stability of the plant. □

**Corollary 2.**  *When the plant meets the stability condition in Theorem 1, the achievable upper bound of $C_{ave}$ under the incremental-coding-based control scheme is given by*

$$\begin{array}{cc} C_{ave} & =\frac{a^{2n}-4^{n}}{n4^{\left(n-1\right)}\left(a^{2}-4\right)}{\left(\frac{\left(a^{n}-1\right)2^{n-1}}{\left(2^{n-1}-a^{n}\right)\left(a-1\right)}w_{max}\right)}^{2}\\ \; & \;+\left(\frac{a^{2n}-1}{n(a^{2}-1)}-\frac{1}{a^{2}-1}\right)\sigma_{w}^{2}. \end{array}$$

**Proof.**  As the plant meets the stability condition in Theorem 1, we have $\frac{a^{n}}{2^{n-1}}<1$. Then, based on the update of $H_{k}$ in Equation (32), we have

(33) $$\lim_{k\to \infty }H_{k}=\frac{\left(a^{n}-1\right)2^{n-1}}{\left(2^{n-1}-a^{n}\right)\left(a-1\right)}w_{max}.$$

As a result, according to Lemma 4, the limit of the achievable upper bound of $C_{ave}^{\left(k\right)}$ is given by

(34) $$\lim_{k\to \infty }C_{ave}^{\left(k\right)}\leq \frac{\left(a^{n}-1\right)2^{n-1}}{\left(2^{n-1}-a^{n}\right)\left(a-1\right)}w_{max}.$$

Based on Equation (26), the right-hand side of Equation (34) is also the achievable upper bound of $C_{ave}$. □

### 3.4. Group Incremental Coding with Finite Blocklength

We omitted the effect of channel coding in the previous analysis. However, considering that incremental coding requires splitting data packets, the impact of different blocklengths in channel coding needs to be considered. According to the conclusions of finite-blocklength coding [37], the achievable transmission rate increases with the coding blocklength. In other words, if incremental coding uses a shorter coding blocklength, the efficiency of the channel coding will be relatively reduced. Therefore, we need to strike a balance between the gains (the average LQG cost) and the disadvantages (the channel-coding efficiency) of incremental coding. This issue can be addressed by employing incremental coding at different resolutions.

From a unified perspective, if we term the current incremental coding as “incremental coding with the minimum resolution of 1 bit” and label the zero-hold strategy as “incremental coding with the maximum resolution of n−1 bits”, we can then explore decoding at various resolutions. For instance, if we split the complete data packet into units of *k* bits, it is equivalent to updating the control strategy every *k* time slots. With a smaller value of *k*, we can achieve a smaller average control cost, but at the same time the channel-coding efficiency will be reduced, and the decoding results and the actuator will need to be updated at a higher frequency. Depending on the practical scenario and constraints, an appropriate resolution *k* should be chosen while maintaining acceptable control costs.

## 4. Applications in Demand-Response Management of Smart Grids

In this section, we extend the single-user control scenario to the case of the multi-user control scenario. A specific multi-user control scenario in demand-response management of smart grids is studied. In this scenario, the cumulative MSE between the electricity load and the supply is formulated as the LQG control cost. On this basis, both the centralized and the distributed communication modes for optimization of the LQG control performance are considered. Under these two communication modes, the joint optimization of source coding and incremental-coding-based control is introduced for the minimization of the LQG control cost.

### 4.1. Multi-User Control Scenario in Demand-Response Management

**Figure 3:**
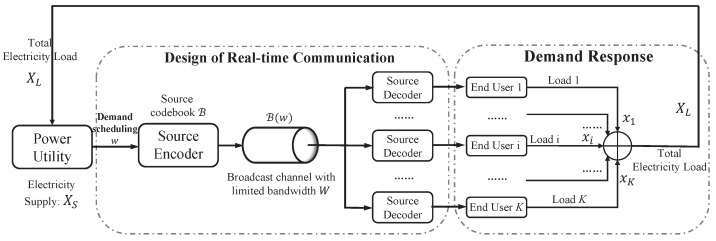
Communication design for real-time signaling transmission in demand-response management of smart grids.

As shown in Figure 3, the aim of DRM is to balance the total electricity load $X_{L}$ of the end users and the electricity supply $X_{S}$ of the power utility, as quickly as possible. However, due to the limited bandwidth of wireless channels and the huge number of end users, emergency-demand scheduling information is difficult to support. As a result, to balance the electricity load and supply in real-time demand response, we introduce joint optimization of the source-codebook design and incremental-coding-based control for the minimization of cumulative MSE between the electricity load and the supply.

At the beginning of each duration *T*, the power utility sends the demand scheduling information ω to the end users according to the total power supply $X_{S}$. The information ω is coded into a binary codeword B(ω) by the source encoder and transmitted to the end users through the wireless channel. However, due to the limited data-transmission power, the wireless channel can only support one bit of error-free transmission per channel use. The duration of each channel use is denoted by one period and is equal to the inverse of the channel bandwidth *W*, i.e., 1/W. As a result, the binary codeword B(ω) is received successively by the end users. Let $B^{\left(n\right)}\left(\omega \right)$ denote the first *n* segment of codeword B(ω). Therefore, the received partial codeword at the *n*-th period of each duration is also $B^{\left(n\right)}\left(\omega \right)$.

For end user *i*, there is a concave utility function $U_{i}\left(x\right)$ to describe the gain that end user *i* obtains from its electricity load *x*, where $U_{i}^{\prime }\left(0\right)=+\infty$. Based on the received partial codeword $B^{\left(n\right)}\left(\omega \right)$ and the utility function $U_{i}\left(x\right)$, the end user *i* adjusts its electricity load in real time. Let $x_{i}\left(t\right)$ denote the real-time electricity load of user *i*, which is determined by the received partial codeword and the utility function. As a result, the total real-time electricity load $X_{L}\left(t\right)$ of all the users is given by

(35) $$X_{L}\left(t\right)=\sum_{i=1}^{K}x_{i}\left(t\right).$$

To balance the total electricity load and the electricity supply of the power utility as quickly as possible, the instantaneous cost-of-demand response is defined as the square error between real-time load $X_{L}\left(t\right)$ and supply $X_{S}$. Let $C_{ave}$ denote the expected cumulative cost in the duration *T*, which is given by

(36) $$C_{ave}=E_{X_{S}∼p}\left\{\int_{0}^{T}{\left(X_{L}\left(t\right)-X_{S}\right)}^{2}dt\right\}.$$

Furthermore, the expected cumulative cost $C_{ave}$ can also be presented as the LQG control cost of the linear control system, in which T=n, a=0, $u_{kn+i}=X_{L}\left(\left(kn+i\right)T_{s}\right)$, and $w_{kn+t}=X_{S}$.

In addition to the balance of electricity load and supply, we should also consider the maximization of the users’ utility under the given electricity-supply constraint. As a result, the users’ demand response $\left\{x_{1}\left(t\right),x_{2}\left(t\right),\cdots ,x_{K}\left(t\right)\right\}$ is expected to converge to the optimal solution to the following problem, as *t* approaches *T*:

(37) $$\begin{array}{cc} \max_{\left\{x_{1},x_{2},\cdots ,x_{K}\right\}} & \;\sum_{i=0}^{K}U_{i}\left(x_{i}\right)\\ s.t.\;\; & \;\sum_{i=0}^{K}x_{i}\leq X_{S},\\ \; & \;x_{i}\geq 0,\;\;\;\;\forall \;i=1,2,\cdots ,K. \end{array}$$

To minimize $C_{ave}$ in Equation (36) while maximizing the total users’ utility in problem (37), both the demand-scheduling signaling and its corresponding source coding shall be carefully designed. In the remainder of this paper, we shall try different schemes to design these parameters and compare their performance in the numerical results.

### 4.2. Demand-Response Management under Distributed-Communication Mechanism

In this subsection, we assume that the power utility has knowledge of all the users’ utility functions. Based on this assumption, the power utility transmits the information of $x_{k}^{*}$ to each individual end user *k* under the distributed-communication mode.

More specifically, the power utility first obtains the optimal solution $\left\{x_{1}^{*},x_{2}^{*},\cdots ,x_{K}^{*}\right\}$ to problem (37). Then, the demand-scheduling information is set to be $\left\{x_{1}^{*},x_{2}^{*},\cdots ,x_{K}^{*}\right\}$ and is transmitted to the end users for adjusting the electricity load. In the remainder of this section, we shall study how to transmit the demand-scheduling information $\left\{x_{1}^{*},x_{2}^{*},\cdots ,x_{K}^{*}\right\}$ over the wireless channel with limited bandwidth *W* to reduce $C_{ave}$ as much as possible.

#### 4.2.1. Sharing the Wireless Channel through TDMA

**Figure 4:**
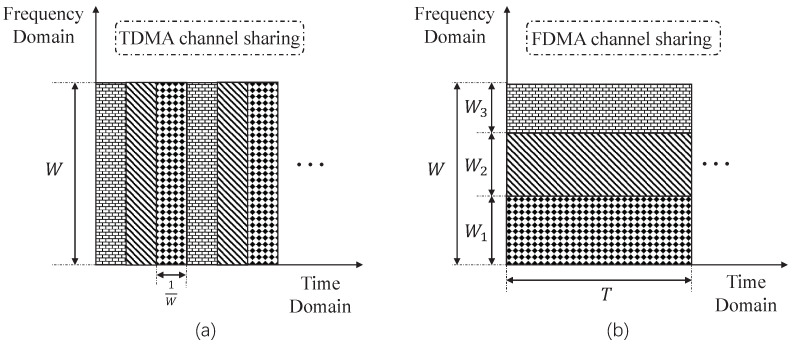
Schematic of sharing the broadcast channel through TDMA or the FDMA mechanism. (**a**) TDMA channel sharing. The channel cycles through the three users, transmitting one bit per time slot of duration 1/W. (**b**) FDMA channel sharing. The channel is shared by three users in the frequency domain.

One straightforward way to transmit demand-scheduling information to end users is by sharing the broadcast channel via TDMA. More specifically, for each individual user *i*, its optimal demand response $x_{i}^{*}$ is determined by the power supply $X_{S}$ from the power utility. Define $f_{i}$ as a mapping from $X_{S}$ to $x_{i}^{*}$. As $X_{S}\in X=\left\{X_{1},X_{2},\cdots ,X_{n}\right\}$, based on $f_{i}$ we can also obtain the alphabet $X_{i}$ for $x_{i}^{*}$ as $X_{i}=\left\{f_{i}\left(X_{1}\right),f_{i}\left(X_{2}\right),\cdots ,f_{i}\left(X_{n}\right)\right\}$. Given the alphabet $X_{i}$ for user *i*, the scheduling information $x_{i}^{*}$ is coded independently as the codeword $B_{i}\left(x_{i}^{*}\right)$ according to a designed codebook $B_{i}$. Then, the codeword $B_{i}\left(x_{i}^{*}\right)$ is transmitted to user *i* over the broadcast channel with bandwidth *W*. The channel is shared equally among all the users in the time domain. In each time slot with duration 1/W, it supports one bit of error-free transmission, as shown in Figure 4a.

Under the TDMA framework, each user *i* receives one new bit of codeword $B_{i}\left(x_{i}^{*}\right)$ every K/W seconds. Then, user *i* adjusts its real-time electricity load $x_{i}\left(t\right)$ according to the received partial codeword $B_{i}^{\left(n\right)}\left(x_{i}^{*}\right)$, where $B_{i}^{\left(n\right)}\left(x_{i}^{*}\right)$ is the first *n* segment of codeword $B_{i}\left(x_{i}^{*}\right)$. As a result, the real-time electricity load of user *i* is given by

(38) $$x_{i}\left(t\right)=\left\{\begin{array}{ccc} w\left(B_{i}^{\left(n\right)}\left(x_{i}^{*}\right)\right), & \; & \frac{nK}{W}\leq t\leq \frac{(n+1)K}{W},\;\;n<l_{x_{i}^{*}},\\ x_{i}^{*}, & \; & t\geq \frac{l_{x_{i}^{*}}K}{W}, \end{array}\right.$$

where *n* is a non-negative integer, variable $l_{x_{i}^{*}}$ is the length of codeword $B_{i}\left(x_{i}^{*}\right)$, and function $w\left(.\right)$ denotes the decision-making scheme for adjusting the real-time electricity load according to the received partial codeword.

To balance the electricity load and supply, the real-time load $x_{i}\left(t\right)$ of user *i* is expected to approach $x_{i}^{*}$ as much as possible. We denote the cumulative MSE between $x_{i}\left(t\right)$ and $x_{i}^{*}$ as $\delta_{i}$, which is given by

(39) $$\begin{array}{cc} \delta_{i} & =\int_{0}^{T}{\left(x_{i}\left(t\right)-x_{i}^{*}\right)}^{2}dt,\\ \; & =\frac{K}{W}\sum_{n=0}^{WT/K}{\left(w\left(B_{i}^{\left(n\right)}\left(x_{i}^{*}\right)\right)-x_{i}^{*}\right)}^{2},\\ \; & =\frac{K}{W}\sum_{n=0}^{l_{x_{i}^{*}}}{\left(w\left(B_{i}^{\left(n\right)}\left(x_{i}^{*}\right)\right)-x_{i}^{*}\right)}^{2}. \end{array}$$

Our aim is to minimize the expectation of $\delta_{i}$ to balance the electricity load and supply as quickly as possible. As the number *K* of users and bandwidth *W* are constant, based on Equation (39) we only need to minimize the expectation of $\sum_{n=0}^{l_{x_{i}^{*}}}{\left(w\left(B_{i}^{\left(n\right)}\left(x_{i}^{*}\right)\right)-x_{i}^{*}\right)}^{2}.$ To this end, we introduce the joint optimization of the source codebook $B_{i}$ and the real-time decision-making scheme w(.), which can be formulated as the following problem:

(40) $$\min_{B_{i},\;w\left(.\right)}\;E_{x_{i}^{*}}\left\{\sum_{n=0}^{l_{x_{i}^{*}}}{\left(w\left(B_{i}^{\left(n\right)}\left(x_{i}^{*}\right)\right)-x_{i}^{*}\right)}^{2}\right\}.$$

It is not a trivial work to solve problem (40) directly, as the feasible numbers of codebook $B_{i}$ and decision-making scheme w(.) are huge. Fortunately, this problem is a special case of problem (7) formulated in our previous work [32]. To solve this problem, a dynamic-programming algorithm is presented (Algorithm 2 in [32]), based on which we can efficiently obtain the optimal source codebook and decision-making scheme in problem (40). Let $\xi_{i}^{*}$ denote the optimal objective value of problem (40). Then, under the optimal source codebook and TDMA, the total cumulative MSE of all users, i.e., $\delta_{TDMA}$ is given by

(41) $$\begin{array}{cc} \delta_{TDMA} & =E\left\{\sum_{i=1}^{K}\delta_{i}\right\}=\frac{K}{W}\sum_{i=1}^{K}\xi_{i}^{*}. \end{array}$$

The careful reader may find that the metric $\delta_{TDMA}$ is different from the metric $C_{ave}$ in Equation (36). We would mention that $KE\left\{\sum_{i=1}^{K}\delta_{i}\right\}$ is an upper bound of $C_{ave}$. This is because

(42) $$\begin{array}{cc} \int_{0}^{T}{\left(X_{L}\left(t\right)-X_{S}\right)}^{2}dt & =\int_{0}^{T}{\left(\sum_{i=1}^{K}\left(x_{i}\left(t\right)-x_{i}^{*}\right)\right)}^{2}dt,\\ \; & \leq \int_{0}^{T}K\sum_{i=1}^{K}{\left(x_{i}\left(t\right)-x_{i}^{*}\right)}^{2}dt,\\ \; & =K\sum_{i=1}^{K}\delta_{i}. \end{array}$$

As *K* and *W* are constant, we can minimize the upper bound of $C_{ave}$ by minimizing the result of $\delta_{TDMA}$.

#### 4.2.2. Sharing the Wireless Channel through FDMA

In this part, we consider sharing the broadcast channel among the users through the FDMA mechanism. By implementing the FDMA mechanism, the information of $\left\{x_{1}^{*},x_{2}^{*},\cdots ,x_{K}^{*}\right\}$ can be transmitted simultaneously to the users. On the other hand, the bandwidth allocation in FDMA provides a more flexible way for resource allocation to minimize the total cumulative MSE.

More specifically, let $\left\{W_{1},W_{2},\cdots ,W_{K}\right\}$ denote the bandwidth allocation for the end users. For an individual end user *i*, it receives a new bit of codeword $B_{i}\left(x_{i}^{*}\right)$ every $1/W_{i}$ seconds. Therefore, under the FDMA mechanism with the optimal source codebook in problem (40), the total cumulative MSE of all the users under the FDMA mechanism, i.e., $\delta_{FDMA}$ is given by

(43) $$\delta_{FDMA}=\sum_{i=1}^{K}\frac{\xi_{i}^{*}}{W_{i}}.$$

In the following, we aim to minimize $\delta_{FDMA}$ by bandwidth allocation. The problem can be formulated as follows:

(44) $$\begin{array}{cc} \min_{W_{1},W_{2},\cdots ,W_{K}} & \;\sum_{i=1}^{K}\frac{\xi_{i}^{*}}{W_{i}}\\ s.t.\;\; & \;\sum_{i=1}^{K}W_{i}=W,\\ \; & \;W_{1},W_{2},\cdots ,W_{K}\geq 0. \end{array}$$

**Theorem 2.**  The optimal bandwidth allocation in FDMA for each individual end user i is $W_{i}^{*}$, which is given as follows:

(45) $$W_{i}^{*}=\frac{W\sqrt{\xi_{i}^{*}}}{\sum_{j=1}^{K}\sqrt{\xi_{j}^{*}}}.$$

**Proof.**  First of all, the objective function $\sum_{i=1}^{K}\frac{\xi_{i}^{*}}{W_{i}}$ of problem (44) is a convex function for $W_{1},W_{2},\cdots ,W_{K}\geq 0$. The constraints of problem (44) are all linear constraints. Therefore, the strong duality condition of problem (44) is established. Then, we can find the optimal solution $W_{i}^{*}$ by minimization of the following Lagrangian function:

(46) $$F\left(W_{i},\lambda_{i},\mu \right)=\sum_{i=1}^{K}\frac{\xi_{i}^{*}}{W_{i}}+\mu \left(\sum_{i=1}^{K}W_{i}-W\right)\;\;-\;\;\sum_{i=1}^{K}\lambda_{i}W_{i}.$$

Based on the strong-duality condition, we have $\frac{\partial F}{\partial W_{i}^{*}}=0$. Then, we can ascertain that

(47) $$-\frac{\xi_{i}^{*}}{W_{i}^{*2}}+\mu^{*}-\lambda_{i}^{*}=0.$$

Furthermore, based on the KKT condition, we have $\lambda_{i}^{*}W_{i}^{*}=0$. As a result, we obtain the relationship between $W_{i}^{*}$ and $\mu^{*}$:

(48) $$W_{i}^{*}=\sqrt{\frac{\xi_{i}^{*}}{\mu^{*}}}.$$

By substituting Equation (48) into Equation (44.b), we finally obtain

(49) $$W_{i}^{*}=\frac{W\sqrt{\xi_{i}^{*}}}{\sum_{j=1}^{K}\sqrt{\xi_{j}^{*}}}.$$

□

**Corollary 3.**  The total cumulative MSE of all users, i.e., $\delta_{FDMA}$ with the optimal bandwidth allocation $W_{i}^{*}$ is given by

(50) $$\delta_{FDMA}=\frac{{\left(\sum_{i=1}^{K}\sqrt{\xi_{i}^{*}}\right)}^{2}}{W}.$$

**Proof.**  By substituting the result of $W_{i}^{*}$ into Equation (43), we can obtain Equation (50). □

**Corollary 4.**  With the optimal bandwidth allocation $W_{i}^{*}$ in FDMA, the total cumulative MSE of all users, i.e., $\delta_{FDMA}$ shall satisfy

(51) $$\delta_{FDMA}\leq \delta_{TDMA}.$$

**Proof.**  The proof of inequality (51) is equivalent to show that the following inequality (52) is established:

(52) $$\frac{{\left(\sum_{i=1}^{K}\sqrt{\xi_{i}^{*}}\right)}^{2}}{W}\leq \frac{K}{W}\sum_{i=1}^{K}\xi_{i}^{*}.$$

Then, inequality (52) can be transformed into the form of the Cauchy–Buniakowsky–Schwarz inequality as follows:

(53) $${\left(\sqrt{\xi_{1}^{*}}+\cdots +\;\;\sqrt{\xi_{K}^{*}}\right)}^{2}\leq \underset{K}{\underset{︸}{\left(1+\cdots +1\right)}}\left(\xi_{1}^{*}+\cdots +\xi_{K}^{*}\right).$$

The equal sign holds if and only if $\xi_{1}^{*}=\xi_{2}^{*}=\cdots =\xi_{K}^{*}$. □

### 4.3. Demand-Response Management under Centralized Communication Mechanism

In this subsection, the demand-scheduling information is set to be the electricity price. According to the information of the electricity price and the utility function, the end users adjust the electricity load to maximize their own benefit, which is a special case of competitive Markov decision processes [38]. On this basis, demand response is managed under a centralized-communication mechanism.

#### 4.3.1. Control with Knowledge of Utility Function

In this part, we still assume that the power utility has knowledge of all the users’ utility functions. Based on these utility functions, the power utility shall decide the electricity price and transmit it to the end users. For a given electricity price *p*, the benefit $b_{i}$ of user *i* is given by

(54) $$b_{i}=U_{i}\left(x_{i}\right)-px_{i},$$

where $x_{i}$ is the electricity load of user *i*.

In the following, we shall study whether there exists an optimal electricity price $p^{*}\left(X_{S}\right)$ for a given power supply $X_{S}$. Based on the information of $p^{*}\left(X_{S}\right)$, each user *i* adjusts the electricity load to the optimal electricity load $x_{i}^{*}$ (the optimal solution to problem (37)) by maximizing its own benefit $b_{i}$. If such $p^{*}\left(X_{S}\right)$ exists, then we shall consider how to transmit it through the broadcast channel.

**Lemma 5.**  There is an optimal electricity price $p^{*}\left(X_{S}\right)$, which is the solution λ to the following equation:

(55) $$\sum_{i=1}^{K}{U_{i}^{\prime }}^{-1}\left(\lambda \right)=X_{S},$$

*where function ${U_{i}^{\prime }}^{-1}\left(.\right)$ is the inverse of the derivative of the utility function $U_{i}\left(.\right)$.*

**Proof.**  As the utility function $U_{i}\left(.\right)$ is a concave function, problem (37) is a convex problem. The Lagrangian function corresponding to problem (37) is given by

(56) $$L\left(x_{i},\lambda_{i},\lambda \right)=-\sum_{i=0}^{K}U_{i}\left(x_{i}\right)+\lambda \left(\sum_{i=1}^{K}x_{i}-X\right)\;\;-\;\;\sum_{i=1}^{K}\lambda_{i}x_{i}.$$

Based on the strong-duality condition, we have $\frac{\partial L}{\partial x_{i}^{*}}=0$. Then, we can obtain

(57) $$-U_{i}^{\prime }\left(x_{i}^{*}\right)+\lambda^{*}-\lambda_{i}^{*}=0.$$

On the other hand, based on the KKT condition, we have $\lambda_{i}^{*}x_{i}^{*}=0$ and $\lambda^{*}\geq 0$. As $U_{i}^{\prime }\left(0\right)=+\infty$, we have $\lambda_{i}^{*}=0$ and $U_{i}^{\prime }\left(x_{i}^{*}\right)=\lambda^{*}$. Furthermore, as utility function $U_{i}\left(.\right)$ is a concave function, its derived function $U_{i}^{\prime }\left(.\right)$ is monotonically decreasing, and the inverse of $U_{i}^{\prime }\left(.\right)$ exists. Therefore, the optimal electricity load $x_{i}^{*}$ can be presented by

(58) $$\left\{\begin{array}{c} U_{i}^{\prime }\left(x_{i}^{*}\right)=\lambda^{*},\\ \sum_{i=1}^{K}{U_{i}^{\prime }}^{-1}\left(\lambda^{*}\right)=X_{S}. \end{array}\right.$$

If the electricity price $p^{*}\left(X_{S}\right)$ is set to be $\lambda^{*}$ in Equation (58), the benefit $b_{i}$ of user *i* is $U_{i}\left(x_{i}\right)-\lambda^{*}x_{i}$. Therefore, for maximization of $b_{i}$, user *i* shall adjust its electricity load $x_{i}$ to satisfy $U_{i}^{\prime }\left(x_{i}\right)=\lambda^{*}$, i.e., $x_{i}=x_{i}^{*}$. □

In the following, we shall design a source codebook B for transmitting the information of the optimal electricity price $p^{*}$ under the incremental-coding mechanism. The aim of incremental coding is minimization of $C_{ave}$. For the end users, they estimate the electricity price as ${\hat{p}}^{\left(n\right)}=w\left(B^{\left(n\right)}\left(p^{*}\right)\right)$ in real time, based on the currently received partial codeword $B^{\left(n\right)}\left(p^{*}\right)$. According to the estimated price $\hat{p}$, each user *i* adjusts its electricity load ${\hat{x}}_{i}^{\left(n\right)}={U_{i}^{\prime }}^{-1}\left({\hat{p}}^{\left(n\right)}\right)$ to maximize its benefit $b_{i}$. Therefore, we can minimize $C_{ave}$ by optimizing codebook B and decision-making scheme w(.) as follows:

(59) $$\min_{B,\;w(.)}\;E_{p^{*}}\left\{\frac{1}{W}\sum_{n=0}^{l_{p^{*}}}{\left(\sum_{i=1}^{K}{U_{i}^{\prime }}^{-1}\left(w\left(B^{\left(n\right)}\left(p^{*}\right)\right)\right)-X_{S}\right)}^{2}\right\},$$

where $l_{p^{*}}$ is the codeword length of realization $p^{*}$. Fortunately, this problem is still a special case of problem (7) in our previous work [32]. Therefore, it can also be solved by a dynamic-programming algorithm, i.e., Algorithm 2 in [32].

#### 4.3.2. Control without Knowledge of Utility Function

In this part, a more practical scenario is considered, in which the power utility has no knowledge of the users’ utility functions. Then, although the optimal electricity price $p^{*}\left(X_{S}\right)$ exists, the power utility cannot obtain it directly.

As a result, we need to design a new demand-scheduling signaling and its corresponding communication mechanism. Based on Lemma 5, we note that the optimal electricity price $p^{*}\left(X_{S}\right)$ is exactly the solution to Equation (55). Fortunately, the function $\sum_{i=1}^{K}{U_{i}^{\prime }}^{-1}\left(.\right)$ is monotone decreasing. This indicates to us that we can find $p^{*}\left(X_{S}\right)$ through a binary search.

More specifically, there are two initial parameters, i.e., the upper bound $p^{up}$ of the electricity price and the lower bound $p^{low}$ of the electricity price, which satisfy

(60) $$\left\{\begin{array}{c} \sum_{i=1}^{K}{U_{i}^{\prime }}^{-1}\left(p^{low}\right)\geq \max\left\{X_{S}|X_{S}\in X\right\},\\ \sum_{i=1}^{K}{U_{i}^{\prime }}^{-1}\left(p^{up}\right)\leq \min\left\{X_{S}|X_{S}\in X\right\}. \end{array}\right.$$

Based on the initial parameters $p^{up}$ and $p^{low}$, the electricity price *p* is defined to be $(p^{low}+p^{up})/2$. According to the electricity price *p*, the users adjust their electricity load to maximize their benefits. For a given price *p*, the total electricity load of all the users’ demand response, $X_{L}$, is given by

(61) $$X_{L}=\sum_{i=1}^{K}{U_{i}^{\prime }}^{-1}\left(p\right).$$

The power utility has the knowledge of $X_{L}$. If $X_{L}>X_{S}$, then the power utility sends bit ‘0’ to the users, to indicate the update of $p^{low}=\left(p^{low}+p^{up}\right)/2$. If $X_{L}<X_{S}$, then the power utility sends bit ‘1’ to the users, to indicate the update of $p^{up}=\left(p^{low}+p^{up}\right)/2$. Based on this, the length of interval $\left[p^{low},p^{up}\right]$ shrinks exponentially. As $p^{*}$ always belongs to the interval $\left[p^{low},p^{up}\right]$, the total electricity load $X_{L}$ also converges to $X_{S}$ exponentially.

**Figure 5:**
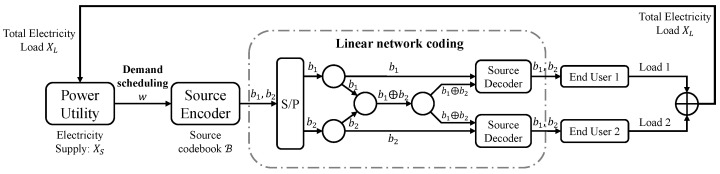
Communication design for real-time signaling transmission with linear network coding. $b_{1}$ and $b_{2}$ denote two consecutive bits after encoding, and ⊕ denotes the exclusive-or operation between the bits.

It is worth mentioning that introducing network coding proves beneficial in multi-receiver multicast scenarios. Take, for instance, the scenario depicted in Figure 5. When dealing with dual receivers, the successful decoding of $b_{1}$ and $b_{2}$ is achieved through a bitwise XOR operation. For example, $b_{1}$ and $b_{2}$ denote two consecutive bits after encoding, and by means of a linear network coding method, the end users can get both bits after decoding. Then we can use incremental coding with a resolution of 2 bits as mentioned at the end of the previous section. The performance analysis in this case can be further investigated in future work.

## 5. Numerical Result

In this section, we present the numerical results to validate our theoretical analysis. In particular, a single-user scenario and a multi-user scenario in smart grids are considered successively in the numerical results.

### 5.1. Single-User Scenario

In the single-user scenario, the parameters of a continuous-time scalar linear control system are set to be $a_{c}=0.8$, $b_{c}=0.5$, $w_{0}=1$, and $x_{0,max}=100$. The wireless bandwidth *W* is set to be 2 kHZ. The time interval 1 ms is normalized as 1. As a result, the parameters of the equivalent discrete-time scalar linear control system are given by a=1.49, b=0.31, and $w_{max}=0.61$.

**Figure 6:**
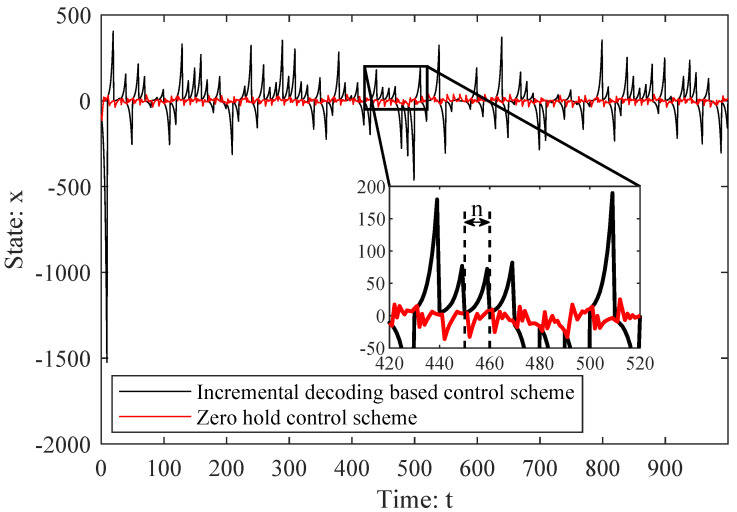
A comparison of zero-hold control and incremental-coding-based control.

In Figure 6, we set the measurement period n=10 and compare the results of the zero-hold control scheme to the incremental-coding-based control scheme in the same stochastic environment. As we can see in Figure 6, state *x* of the incremental-coding-based control scheme is much more stable around 0 than that of the zero-hold control scheme. In particular, state *x* under the zero-hold control scheme shifts away from 0 exponentially over time until a complete codeword is received in the last symbol period. On the other hand, state *x* under the incremental-coding-based control scheme begins to shift to 0 after the first symbol period. This is because the controller performs the control action in real time, based on a partially received codeword. As a result, the incremental-coding-based control scheme shows less LQG control cost than that of the zero-hold control scheme.

**Figure 7:**
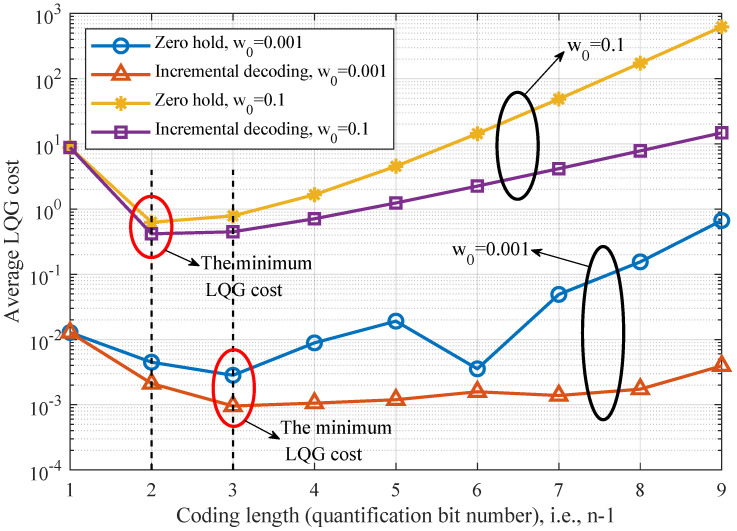
Comparison of LQG control cost under different quantization bit numbers.

In Figure 7, we set the noise factor $w_{0}$ as 0.001 and 0.1, respectively, and show the LQG control cost under different quantization bit numbers. Firstly, as we can see, the LQG control cost of the incremental-coding-based control scheme is less than that of the zero-hold control scheme in all cases of quantization bit numbers. Then, for a given noise factor *w*, there is an optimal quantization bit number that minimizes the LQG control cost. This is because a too-large or too-small number of quantization bits will cause obvious control errors. In particular, a too-small number of quantization bits will cause more quantization error and a too-large number of quantization bits will cause more transmission delay, thereby inducing more noise in the control process. On the other hand, when the noise factor $w_{0}$ is 0.001, the optimal quantization bit number is 3. But when the noise factor $w_{0}$ is 0.1, the optimal quantization bit number is 2. This is because, in cases of smaller noise factor, the quantization error is more important than the noise error. Therefore, a greater quantization bit number should be considered, to reduce the quantization error.

### 5.2. Multi-User Scenario

In this subsection, we consider a multi-user scenario in the demand response of smart grids. We set the total power supply at the end of the power utility, $X_{S}$, to be an independent and identically distributed random variable with values drawn from the set X={4,5,6,7,8,9,10}. The associated discrete probability distribution is represented by p={0.1,0.1,0.2,0.3,0.15,0.05,0.1}. The power utility broadcasts scheduling information to the users, where the number of users is set to K=3. The utility function of each user is set to $U_{i}\left(x_{i}\right)=c_{i}\ln\left(1+x_{i}\right)$, where $c_{1}=c_{2}=2$, $c_{3}=4$. The bandwidth of the wireless channel is *W*, in which the corresponding symbol period $\frac{1}{W}$ is normalized as 1. The duration of state $X_{S}$ is set to be T=20.

**Figure 8:**
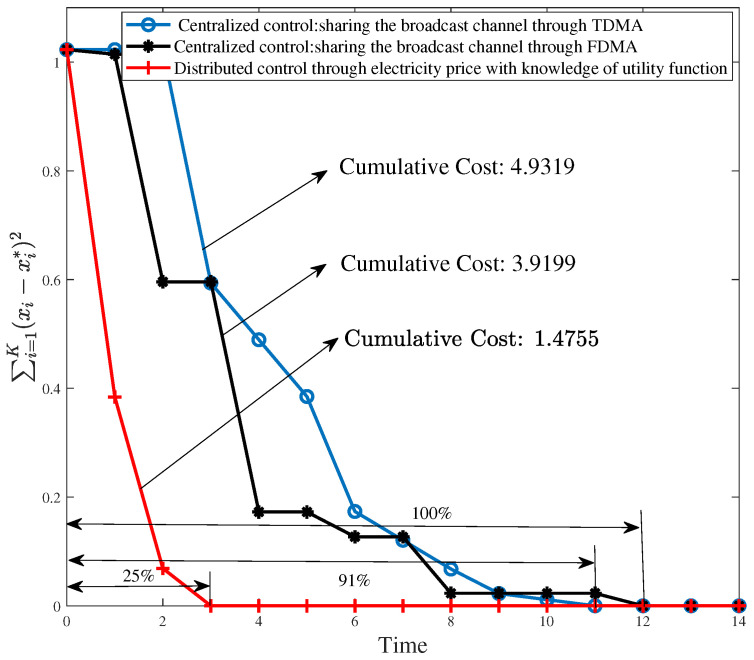
Comparison of demand-response performance under different control mechanisms with incremental coding.

In Figure 8, we compare demand-response performance under different control mechanisms. More specifically, we show the expectation of $\sum_{i=1}^{K}{\left(x_{i}-x_{i}^{*}\right)}^{2}$ in the entire process of scheduling signaling transmission. As we can see in Figure 8, compared to centralized control mechanisms, distributed control through electricity price not only exhibits the least cumulative cost 1.4755, but also reduces the cost to 0 fastest, i.e., using 25% of the time of centralized control with FDMA. On other hand, we can also see that the cumulative cost corresponding to centralized control with FDMA is about 3.9199 and smaller than 4.9319 of TDMA. This is consistent with Corollary 4.

**Figure 9:**
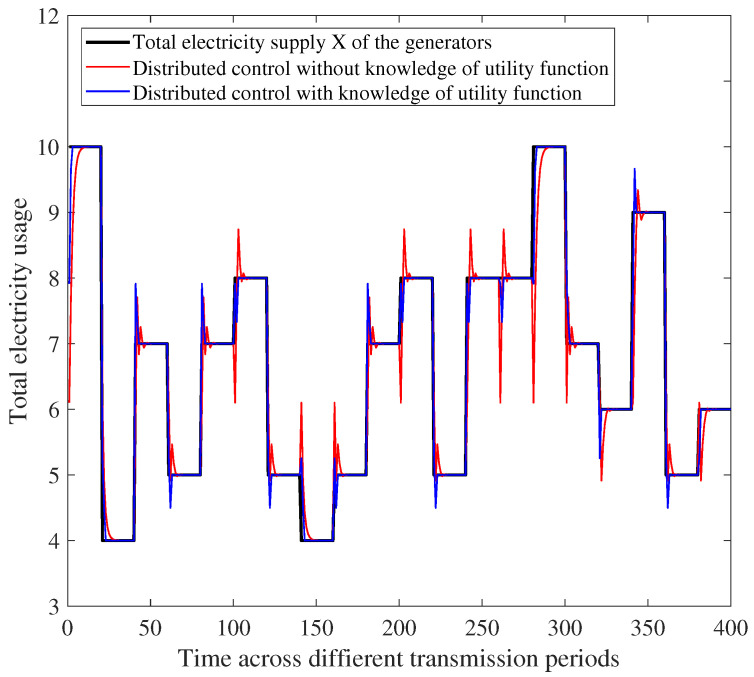
Comparison of demand-response performance under distributed control with or without knowledge of users’ utility.

In Figure 9, under the distributed-control mechanism, we compare the demand-response performance of cases of power utility with or without knowledge of the users’ utility functions. As we have seen, under the distributed-control mechanism, when the power utility has knowledge of all the users’ utility functions, the total electrical load converges to the supply faster than in the case where users’ utility functions are unknown. On the other hand, as a binary search is implemented, the total electricity load converges fast enough in the case that the users’ utility functions are unknown.

## 6. Conclusions

In this paper, we introduced an incremental-coding-based communication mechanism into a remote linear control process over communication networks. Firstly, we considered a linear control system with a single controller and a single plant, and we proposed a control scheme based on incremental coding. Both the stabilization condition of the plant and the average LQG control cost were analyzed under the incremental-coding-based control scheme. The analytical results showed that the proposed incremental-coding-based control scheme significantly outperformed the traditional zero-hold control scheme under the LQG performance measure. Furthermore, we extended the linear control system from a single-user to a multi-user control scenario. A specific multi-user control scenario in demand-response management of smart grids was studied. By introducing the incremental-coding-based communication mechanism with joint optimization of source coding and decision making, the minimization of the performance loss that is induced by the latency of demand scheduling signaling was finally achieved.

## Data Availability

Data are contained within the article.
